# Editorial: Spotlight on aging: physiology, prevention, and management of skeletal muscle atrophy

**DOI:** 10.3389/fphys.2023.1333577

**Published:** 2023-12-05

**Authors:** Gabriel Nasri Marzuca-Nassr, Luis Peñailillo, Denisse Valladares-Ide, Sergio Martinez-Huenchullan, Rui Curi, Sandro Massao Hirabara, Kaio Fernando Vitzel

**Affiliations:** ^1^ Departamento de Ciencias de la Rehabilitación, Universidad de La Frontera, Temuco, Chile; ^2^ Interuniversity Center for Healthy Aging RED21993, Talca, Chile; ^3^ Faculty of Rehabilitation Sciences, Exercise and Rehabilitation Sciences Institute, Universidad Andrés Bello, Santiago, Chile; ^4^ Instituto de Ciencias de la Salud, Universidad de O'Higgins, Rancagua, Chile; ^5^ School of Physical Therapy, Faculty of Medicine, Universidad Austral de Chile, Valdivia, Chile; ^6^ Interdisciplinary Post-graduate Program in Health Sciences, Universidade Cruzeiro do Sul, São Paulo, Brazil; ^7^ School of Health Sciences, College of Health, Massey University, Auckland, New Zealand

**Keywords:** skeletal muscle atrophy, aging, ageing, muscle architecture, sarcopenia

## Introduction

Atrophy of skeletal muscle during aging is a subject of heightened interest for researchers, as the muscle tissue is an essential regulator of whole-body metabolism, bone health, fall prevention, cognitive function, and general wellbeing. Indeed, the precise role of skeletal muscle and strategies to prevent the loss of skeletal muscle mass in individuals aged 60 years or older have been reported (Marzuca-Nassr et al., 2020a; Marzuca-Nassr et al., 2023).

For example, previous studies demonstrated that skeletal muscle atrophy caused by aging can be attenuated/prevented using appropriate therapeutic strategies, including different physical exercise modalities, nutritional supplementation, and medications (Marzuca-Nassr et al., 2020b; Cuyul-Vasquez et al., 2023). However, the most appropriate exercise, supplement, or medication, individually or in combination, remains undefined. Thus, identifying the most effective strategies to attenuate or reverse skeletal muscle atrophy in this particular population is crucial.

Towards this goal, the *Frontiers in Physiology* Healthy Aging series Research Topic Spotlight on Aging: Physiology, Prevention, and Management of Skeletal Muscle Atrophy was created. We encouraged researchers investigating the physiology of skeletal muscle atrophy, with an emphasis on the following subjects: skeletal muscle architecture and mechanical properties in older individuals and/or exercise modalities, oral supplements, medications, or other therapeutic alternatives that prevent and/or manage skeletal muscle atrophy, to submit their Original Research, Systematic Review (with or without Meta-analysis), or any other article type within the scope of the journal, section, and Research Topic for consideration for publication in the Healthy Aging series. It is important to point out that the objective of this Research Topic is in line with the United Nations Decade of Healthy Aging (2021–2030) Sustainable Development Goals, #SDG3, #SDG10, and #SDG11, which collectively seek to improve lives of older people, their families, and communities. Below, we selected several examples of manuscripts that address some of these Research Topic subjects and provide future directions.

Different resistance training modalities, including progressive resistance training (moderate to high-intensity exercises at slow speed) and power training (low to moderate intensity exercises at maximal velocity), are vital strategies to increase skeletal muscle mass and strength within the same resistance training program. Alternatively, Angleri et al. reported that suspension training (multi-planar and multi-joint exercises using hanging straps to suspend body segments, which create an unstable environment and use the body weight against gravity) can also increase muscle mass and strength.

Kemmler et al. also assessed the effects of resistance exercise in older individuals. The authors reported that high-intensity dynamic resistance exercise training (HIT-RT) for 18 months, including supplementation with protein, vitamin D, and calcium, increased lean body mass and skeletal muscle strength (isokinetic leg extension) and reduced total body fat and abdominal fat percentage in sarco-osteopenic men aged 72 years or older. The improvements were most significant after 8 months of training, but small continuous gains were observed until the end of the 18-month trial. This whole-body training regimen was always carried out under professional supervision, using gym equipment, and designed to be time-efficient (two ∼50-min sessions/week). Also, the training was periodized with increasing exercise intensities and varying movement velocities and time under load. This approach indicated that HIT-RT could be a safe and long-term training option for older populations, producing positive changes associated with cardiometabolic health in sarco-osteopenic individuals.

Furthermore, Caparrós-Manosalva et al. assessed the effects of high-intensity interval training (HIIT) resistance exercise training as a potential treatment for sarcopenia. While HIIT is recommended to maintain and increase muscle function, few studies have investigated the interaction between HIIT and muscle mass, strength, and power in a healthy older population, with even fewer assessing the effects on the dominant and non-dominant lower limbs. The authors reported that HIIT promotes increases in lean muscle mass, strength, power, and early rate of force development of the lower limbs in healthy older and young subjects and differences between the dominant and non-dominant limbs. Since asymmetry of muscle function in the lower limbs of older adults is related to an increased risk of falls and functional limitations, the effects of HIIT on lower limb performance must be evaluated.

Another study by Monteiro et al. explored the effects of functional training on the body composition of older people through a systematic review. This strategy has gained popularity among clinicians as an alternative therapeutic option for improving muscle mass during aging because it involves multicomponent exercises performed in different planes, and more importantly, these exercises simulate daily activity. Interestingly, even with the popularity of this type of training, only five studies were included in this review, which highlights the need for more randomized clinical trials in the field. While this study demonstrated that functional training could effectively reduce fat mass and improve lean mass, these improvements were only observed after long training periods (five to six months), with shorter programs (10–12 weeks) yielding conflicting results.

From a slightly different perspective, Lim et al. investigated the effects of community-based exercise programs, dividing the participants (men and women) according to their self-reported exercise mode and volume on bone health, body composition, and functional performance. They compared four groups of individuals who reported performing aerobic exercises, muscle strengthening exercises, and Tai Chi and a group reporting sedentary behavior. The weekly load was categorized into minimum, low, moderate, and high exercise volume. The authors found that bone density was greater in those who performed strengthening exercises compared to sedentary individuals. Additionally, those who performed high volumes of exercise exhibited more favorable changes in body composition. All three levels of exercise were associated with lower body fat and higher lean body mass than sedentary participants, while no changes in bone health were reported. The authors concluded that health risks can be mitigated by participating in different community-based exercise modalities, but increased exercise volume or load may be needed to improve bone health.

Lastly, Reis-Silva et al. performed a meta-analysis review to evaluate the effects of whole-body vibration exercise (WBVE) on body composition as a physical exercise modality recommended for people over 60, an age when there are changes in body composition, including a decrease in skeletal muscle mass and an increase in fat mass. The authors included eight studies evaluating the effects of WBVE on the skeletal muscle of older individuals; however, none reported significant improvements in body composition or overall changes in fat, lean, or skeletal muscle mass. Thus, there is currently no evidence that WBVE can benefit the body composition of older men and women.

## Future directions

Future research in aging muscle atrophy has to consider the patient’s health condition, including associated diseases, the patient’s capacity to perform specific physical exercise training, the best physical exercise modality, and the most appropriate dietary supplement or medication. The prevention of skeletal muscle mass loss due to aging must be addressed using a combination of physical exercise training and nutritional supplementation from different sources. The emerging interest in preventing and managing skeletal muscle atrophy in the aging population will likely lead to effective therapeutic strategies. The abovementioned issues should be evaluated in long-term studies with large sample sizes to define clinical procedures and apply them to the public health sector.
